# Iatrogenic Vessel Dissection in Endovascular Treatment of Acute Ischemic Stroke

**DOI:** 10.1007/s00062-017-0639-z

**Published:** 2017-11-02

**Authors:** Barbara Goeggel Simonetti, Justine Hulliger, Etienne Mathier, Simon Jung, Urs Fischer, Hakan Sarikaya, Johannes Slotboom, Gerhard Schroth, Pasquale Mordasini, Jan Gralla, Marcel Arnold

**Affiliations:** 1 Department of Neurology, Inselspital, University Hospital Bern, University of Bern, Bern, Switzerland; 2 Ospedale San Giovanni Bellinzona, Bellinzona, Switzerland; 3 Department of Neurology, University Hospital Zürich, Zürich, Switzerland; 4 Department of Diagnostic and Interventional Neuroradiology, Inselspital, University Hospital Bern, University of Bern, Freiburgstraße 10, 3010 Bern, Switzerland

**Keywords:** Iatrogenic, Dissection, Endovascular treatment, Ischemic stroke, Interventional neuroradiology

## Abstract

**Purpose:**

Knowledge about the localization and outcome of iatrogenic dissection (ID) during endovascular treatment of acute ischemic stroke (AIS) is limited. We aimed to determine the frequency, clinical aspects and morphology of ID in endovascular AIS treatment and to identify predictors of this complication.

**Methods:**

Digital subtraction angiography (DSA) of ID carried out during endovascular treatment between January 2000 and March 2012 have been re-evaluated. The ID localization and morphology were analyzed and related to the interventional techniques. Baseline clinical and radiological findings, treatment modality and outcome were compared with patients without ID.

**Results:**

Out of 866 patients 18 (2%) suffered an ID (44% female, median age 64 years). Localization was extracranial in 15 (83%, 14 internal carotid artery and 1 vertebral artery) and intracranial in 3 (17%; 1 vertebrobasilar dissection and 2 in the anterior circulation). Of the IDs 5 (28%) resulted in a high-degree, 3 (17%) in a moderate, 5 (28%) in a mild and 5 (28%) in no stenosis and 8 IDs were stented in the acute phase. At 3 months 7 (42%) patients had a favorable outcome (modified Rankin score mRS ≤ 2) and 6 (33%) patients had died. Patients with ID had a different stroke etiology (*p* = 0.041), were more likely to be smokers (44% versus 19%, *p* = 0.015) and were more likely to be treated with mechanical thrombectomy (100% versus 60%, *p* < 0.001). Although two ID patients had relevant complications, the outcome did not differ between the groups.

**Conclusion:**

The occurrence of ID is a rare complication of endovascular AIS treatment associated with smoking and mechanical thrombectomy.

**Electronic supplementary material:**

The online version of this article (10.1007/s00062-017-0639-z) contains supplementary material, which is available to authorized users.

## Introduction

Acute arterial ischemic stroke (AIS) is one of the leading causes of mortality and morbidity worldwide [1, 2]. Immediate and efficacious revascularization of the occluded vessel is crucial to improve outcome [3, 4]. Over the past decade endovascular management of AIS has rapidly improved and several catheter-based treatment systems have been developed. To date, 5 randomized controlled trials have shown that endovascular clot retrieval in addition to best medical treatment with or without intravenous recombinant tissue plasminogen activator (rtPA) improves outcome in patients with stroke occurring in the anterior circulation with proximal vessel occlusion [5–9]; however, despite minimally invasive device systems, endovascular procedures still carry a risk of iatrogenic complications, potentially worsening the outcome. Manipulation of the catheter, guidewire or thrombectomy devices could cause vessel wall injury. Studies reporting ID rates during cerebral angiography mainly include diagnostic angiography only [10–12] or interventional angiography for indications other than AIS, often in an elective, non-urgent setting [13–15]. Only a few studies have reported complication rates in endovascular AIS treatment (ranging from 1.7% to 3.9%), but clinical characteristics, risk factors, outcomes and morphological descriptions are lacking [5, 9, 16, 17]. We aimed to assess the localization and morphology of ID, the clinical characteristics and risk factors for ID and outcomes in patients suffering from ID.

## Methods

Our study is based on the Bernese stroke registry, a prospective database on patients with AIS [18–20]. Baseline and 3‑month outcome data on consecutive AIS patients admitted to the Neurological Department of the University Hospital of Bern are collected. For this study, we reviewed clinical and imaging data on all AIS patients aged ≥16 years who underwent endovascular AIS treatment between January 2000 and March 2012. All images of confirmed or assumed IDs were retrieved from our PACS (picture archiving and communicating system) and the digital subtraction angiography (DSA) series were carefully re-evaluated for this study. An ID was defined as the presence of any of the following signs on DSA: intimal flap, fusiform or irregular aneurysmal dilation at a non-bifurcation site, double lumen or a string-and-bead sign, or if signs of a wall hematoma were present on fat-saturated T1-weighted magnetic resonance imaging (MRI).

Baseline clinical data, such as age, sex, pre-stroke score on the modified Rankin scale (mRS), vascular risk factors, National Institutes of Health Stroke score (NIHSS) on admission, and stroke etiology according to the Trial of Org 10172 in Acute Stroke Treatment (TOAST) classification [21] were collected. Imaging data, such as vessel occlusion site and grade of occlusion, were assessed at baseline, and after endovascular treatment. Furthermore, imaging in ID cases was reviewed for ID characteristics on DSA, such as localization and stenosis caused by an ID in the acute phase DSA and recanalization on follow-up imaging by magnetic resonance angiography (MRA) or computed tomography angiography (CTA). Stenosis caused by an ID was defined as high grade if ≥80% of the vessel lumen was obstructed, moderate for 50–79%, and mild for <50% local degree of stenosis. The length of the stenosis was classified as either short (≤1 cm) or long (>1 cm).

Follow-up imaging was performed in all patients between 12 and 24 h after the intervention or sooner in the case of any clinical deterioration. The remaining local arterial constriction was graded according to the thrombolysis in myocardial ischemia (TIMI) classification [22]. All interventions were performed in our 24-h/7-day stroke unit by 5 fully trained and experienced neurointerventionalists in accordance with institutional guidelines. Endovascular treatment was based on a diagnostic transfemoral 4‑vessel DSA and consisted of (1) mechanical thrombectomy (MT), (2) local intra-arterial thrombolysis using urokinase, (3) combined intra-arterial lysis with urokinase and systemic intravenous thrombolysis using rtPA or (4) by a combination of MT and pharmacological thrombolysis. Until the introduction of stent retriever technology in 2010, MT was performed by aspiration via a 5 French aspiration catheter (Tracker 35, Boston Scientific; VASCO-ASP, BALT [Montmorency, France]), percutaneous transluminal angioplasty (PTA), stenting or gentle mechanical disruption of the thrombus in combination with local injection of urokinase into the thrombus for up to 2 h. The Merci device (Concentric Medical, Mountain View, California, USA) has not been used. Based on the localization, grade of stenosis and hemodynamic relevance of the lesion, the responsible interventional neuroradiologist and neurologist decided on endovascular treatment of ID. Clinical outcome at 3 months was assessed using the mRS, with favorable outcome defined as a mRS score ≤ 2.

### Statistics

Statistical analyses were performed using the SPSS^©^-software version 21. Pearson’s χ^2^-test or Fisher’s exact test (nominal variables) and Mann-Whitney U‑test (ordinal, continuous variables) were used for group comparison of patients with and without ID. A 2-sided probability value *p* < 0.05 was considered statistically significant.

## Results

Out of 869 consecutive AIS patients who underwent an acute endovascular stroke intervention, 866 were included in this study with a median age of 68 years (interquartile range IQR 57–76 years) and 385 (45%) women. Of the patients three had to be excluded from the study because the intervention was not documented in our PACS system.

### Iatrogenic Arterial Dissection

18 patients (2%) suffered from ID during the endovascular procedure. No patient developed multiple IDs. Clinical and radiological characteristics are summarized in Table 1 and 2.

**Table 1:** Clinical and Radiological Characteristics of Patients with ID

Patient no.	Age (years)	Sex	Independent before stroke (mRS 0–2)	Occluded vessel diagnosed in angiography	Type of intervention IA, IV/IA, MT	ID localization	Vessel dissected	Grade of stenosis caused by ID	ID length	ID-specific acute intervention	Pharmacol. treatment	Residual constriction (TIMI) of dissected vessel in 12–24 h control CTA/MRA	3-month follow-up mRS
1	64	M	Yes	1: left ICA ec 2: left MCA M1	MT (thrombus aspiration)	Extracranial	Left ICA	50–80%	Long	No	NS	Only CT	6
2	76	M	Yes	1: right ICA ec 2: right ICA T	MT (thrombus aspiration)	Extracranial	Right ICA	0	Short	No	NS	3	3
3	60	M	Yes	1: left VA 2: BA prox	IA and MT (PTA)	Extracranial	Left VA	<50%	Short	No	NS	0	6
4	64	M	Yes	1: left VA 2: BA prox	IA and MT (thrombus aspiration)	Intracranial	Left VA, BA prox	0	Short	Stent	NS	3	6
5	33	M	Yes	1: left ICA ec 2: left ICA T	IA and MT (thrombus aspiration)	Extracranial	Left ICA	<50%	Long	Stent	NS	3	2
6	65	F	Yes	Left ICA T	IA and MT (thrombus aspiration)	Extracranial	Left ICA	<50%	Long	No	NS	3	3
7	75	F	Yes	Left MCA M1	IA and MT (Solitaire® stent)	Extracranial	Left ICA	<50%	Short	No	OAC	3	1
8	79	M	Yes	1: right ICA ec 2: right MCA M1	IV/IA and MT (Solitaire® stent)	Extracranial	Right ICA	<50%	Short	No	ASA + clopidogrel	3	2
9	79	F	Yes	1: right ICA ec 2: right MCA M1	MT (intracranial stent)	Extracranial	Right ICA	81–99%	Long	Stent	ASA + clopidogrel	2	4
10	71	M	Yes	1: left ICA 2: left MCA M1	MT (thrombus aspiration)	Extracranial	Left ICA	81–99%	Short	Stent	NS	3	6
11	62	M	Yes	1: right ICA ec 2: right MCA M2	IV/IA and MT (thrombus aspiration)	Extracranial	Right ICA	81–99%	Short	Stent	ASA + clopidogrel	3	2
12	45	F	Yes	1: right ICA ec 2: right MCA M1	IV/IA and MT (thrombus aspiration)	Intracranial	Right MCA M1	50–80%	Short	No	NS	3	6
13	45	F	Yes	Right MCA M1	MT (Solitaire® stent)	Extracranial	Right ICA	81–99%	Long	Stent	ASA + clopidogrel	Only CT	1
14	56	M	Yes	Right MCA M1	MT (thrombus aspiration)	Extracranial	Right ICA	81–99%	Long	Stent	OAC	2	1
15	77	F	No	Left MCA M1	MT (Solitaire® stent)	Extracranial	Left ICA	0	Short	No	NS	Only CT	6
16	79	F	Yes	Left ICA T	MT (thrombus aspiration)	Extracranial	Left ICA	50–80%	Long	Stent	OAC	3	4
17	56	F	Yes	Left ICA T	IA and MT (thrombus aspiration and intracranial stent)	Intracranial	Left ICA	0	Short	No	ASA + clopidogrel	3	0
18	62	M	Yes	Left ICA ec + ic	IV/IA and MT (thrombus aspiration, PTA and intracranial stent)	Extracranial	Left ICA	0	Short	No	ASA + clopidogrel	Only CT	3

*F* female, *M* male, *ec* extracranial, *ic* intracranial, *NS* not stated, *ASA* acetylsalicylic acid, *OAC* oral anticoagulation, *MCA* malignant middle cerebral artery, *ICA* internat carotid artery, *VA* vertebral artery, *mRS* modified Rankin scale, *IV* intravenous thrombolysis, *IA* intraarterial, local thrombolysis

**Table 2:** Comparison of patients with and without Iatrogenic Dissection (ID), following endovascular treatment of acute ischemic stroke

Variable	Valid *n* (total *n* = 866)	ID (*n* = 18)	No ID (*n* = 848)	2-sided *p*
Age (years), median (IQR)	866	64 (56–77)	68 (58–76)	0.608
Female	866	8 (44%)	377 (45%)	1.000
Pre-AIS mRS ≤ 2	865	17 (94%)	832 (98%)	0.288
NIHSS, median (IQR)	864	15 (12–19)	15 (10–20)	0.974
Vascular risk factors				
– Diabetes mellitus	864	5 (28%)	120 (14%)	0.163
– Arterial hypertension	864	12 (67%)	521 (62%)	0.808
– Hypercholesterolemia	866	8 (44%)	453 (52%)	0.635
– Current smoking	851	8 (44%)	161 (19%)	0.015
– Past smoking (≤5 years)	851	0	104 (13%)	0.151
– Coronary heart disease	862	1 (6%)	161 (19%)	0.222
– Atrial fibrillation	787	5 (33%)	288 (37%)	1.000
– Previous ischemic cerebrovascular event	866	2 (11%)	133 (16%)	1.000
– Family history: stroke	637	2 (22%)	106 (17%)	0.854
sCAD as AIS etiology	866	2 (11%)	33 (4%)	0.162
Time from symptom onset to intervention (min), median (IQR)	858	272 (228–378)	273 (220–345)	0.566
Mechanical thrombectomy	866	18 (100%)	506 (60%)	<0.001
3-month follow-up				
Favorable outcome (mRS ≤ 2)	837	7 (39%)	349 (43%)	0.814
Mortality	837	6 (33%)	198 (24%)	0.405

*IQR* interquartile range, *AIS* acute ischemic stroke, *mRS* modified Ranking scale, *sCAD* spontaneous cervical artery dissection

**Fig. 1:**
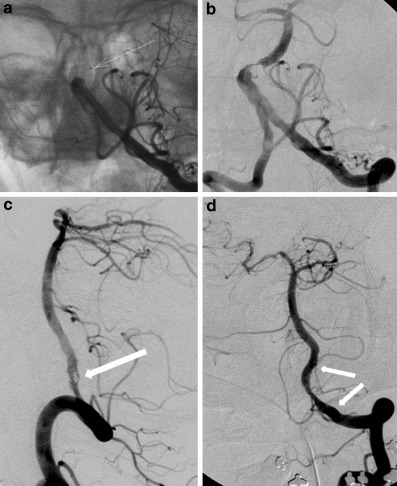
DSA of patient 4, a 64-year-old man with ID at the vertebrobasilar junction. **a** Left VA injection shows the occlusion of the proximal basilar artery with the tip of the microcatheter in the thrombus (*arrow*) during application of urokinase. **b** The underlying high-grade stenosis becomes visible after successful local intra-arterial thrombolysis and was treated by PTA. **c** The dissection following PTA is clearly visible on the DSA in lateral projection (*arrow*). **d** Stabilization of the dissection by insertion of a small self-expanding stent (*arrows*)

15 patients (83% of all IDs) suffered an extracranial ID of which 14 were located in the internal carotid artery (ICA) and 1 in the vertebral artery (VA). The VA dissection was a small, hemodynamically irrelevant intimal flap during passage of the C1-loop of the dominant VA with the 5‑French aspiration catheter. Of the patients 3 (17%) had an intracranial ID, located at the vertebrobasilar junction (Fig. 1), in the distal ICA, and in the middle cerebral artery (M1 branch).

The ID was long in 7 (39%) and short in 11 patients (61%). The stenosis caused by the ID was high grade in 5 (28%), moderate in 3 (17%), and mild in 5 (28%) cases; the remaining 5 (28%) IDs did not result in stenosis. No occlusive ID was observed. Recanalization of ICA occlusion (tandem and carotid-T occlusions) was the most frequent reason for dissections (12 out of 16 in the anterior circulation). Movement of the patient during navigation through the occluded ICA and coiling or kinking of the cervical segment of the ICA most likely caused the IDs in 9 patients. Acute management of the ID by stent placement was performed in 8 patients (44%). All 5 patients with high-grade stenosis were treated as well as 1 patient with moderate, 1 with mild stenosis and 1 patient without stenosis. None of the ID patients showed clinical signs clearly attributable to the ID in the short-term follow-up and new additional ischemic or hemorrhagic lesions were not detected on CT or MRI 12–24 h after endovascular treatment. At 3‑month follow-up, 7 patients had a favorable outcome (mRS ≤ 2) and 6 had died, of which 4 patients died as a result of the AIS as a malignant middle cerebral artery (MCA) infarction or extensive brainstem infarction due to basilar artery (BA) occlusion, without any further signs of ID-related complications. The other 2 patients died from AIS complications, possibly related to the ID as described.

Patient 4 had an extended stroke affecting the cerebellum bilaterally and right thalamus and underwent PTA of a proximal BA stenosis. The vertebrobasilar dissection became visible after aspiration of the occluding thrombus and was treated by implantation of a short stent (Fig. 1). The 12–24 h follow-up MRA revealed a contrast defect at the site of the stent, either caused by an in-stent occlusion or an artefact and 3 days after the initial event, the patient suffered recurrent AIS with new pontine lesions leading to death 1 day later.

Patient 12 had an intracranial MCA-M1 ID after AIS due to a carotid-T occlusion and died 12 days later of an intracranial hemorrhage. The M1 ID was not treated with a stent because the risk of acute endovascular treatment was estimated to be high in a patient with systemic lupus erythematosus under oral corticosteroid treatment. She had been treated with aspirin since the 12–24 h follow-up MRI and MRA showed a complete recanalization of the occluded intracranial ICA and no hemorrhagic or new ischemic lesions.

### Comparison of AIS Patients with and without ID

Most of the clinical and radiological baseline variables in AIS patients treated endovascularly with and without ID did not differ significantly (Table 2). Stroke etiology and percentage of active smokers differed, with ID patients having a cardioembolic etiology less often but an undetermined AIS etiology more often (supplementary Fig. 1) and more often being smokers. Patients with ID were more likely to have been treated with MT than AIS patients without ID. All ID occurred in patients who had undergone MT. Clinical data at 3‑month follow-up were available for all ID and for 819 (97%) of the non-ID patients. Outcome did not differ, either in the distribution of the mRS scores (supplementary Fig. 2) or when dichotomized (*p* = 0.814) or comparing mortality (*p* = 0.405).

## Discussion

The results of our study indicate that the risk for ID in AIS patients undergoing endovascular treatment is low. Mechanical thrombectomy and smoking status are associated with a higher risk for ID. The ID did not have a significant impact on clinical outcome compared to patients without ID despite severe complications possibly related to ID in 2 patients. To date, arterial dissection as a complication of endovascular AIS treatment has been systematically reported by the MR CLEAN, REVASCAT, SWIFT and STAR investigators [5, 9, 16, 17]. Dissection occurred in 1% of STAR patients and 3.9% of REVASCAT patients as a complication of AIS treatment with either Solitaire® (Medtronic, Dublin, Ireland) flow restoration thrombectomy or combined with medical management alone [9, 17]. The MR CLEAN trial found a procedure-related dissection in 1.7% and vessel perforation in 0.9% of patients who received intra-arterial treatment, but provided no detailed information on the type of treatment administered (e.g. intra-arterial thrombolysis, MT or both) [5]. In the SWIFT trial, ID was observed in 5 (3.5%) of the 144 patients treated with MT (Merci® and Solitaire® devices) [16]. These numbers are in line with the ID frequency of 2% (18/866) overall and 3.4% (18/524) seen in MT patients in our study. One of the IDs occurring in SWIFT was adjudicated to be a serious adverse event; however, detailed information on clinical and radiological characteristics and outcome of patients with ID are lacking in all of the abovementioned studies. In a study on interventional cerebral angiographies with indications other than AIS, ID occurred in 0.26–0.7% of patients, all being asymptomatic [13, 15]. In purely diagnostic cerebral angiography, ID occurred in 0–0.4% of patients, and again all were described as being asymptomatic [10–13].

**Fig. 2:**
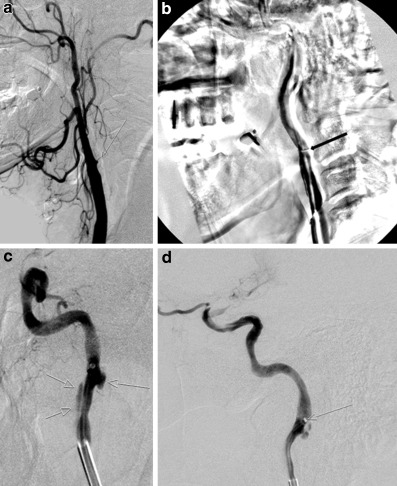
DSA of patient 1, a 64-year-old man with an ICA/MCA tandem occlusion. **a** The stump of the occluded ICA can be identified in the lateral projection of the DSA (*arrow*). **b** Frozen image of the DSA series performed via the aspiration catheter, which has passed the site of occlusion. The original shape of the ICA is subtracted and outlined as white shadow behind the dark course of the ICA after ipsilateral turn of the head. The site of the dissection is clearly visible as a buttonhole stenosis (*arrow*). **c**, **d** The site and extension of the dissection (*arrows*) become visible after initiation of general anesthesia and thrombus aspiration through the 8 F guiding catheter with its tip distal from the site of occlusion

In this study 12 out of 16 dissections in the anterior circulation occurred during recanalization of a former occluded ICA. The fact that this occlusion location is the most difficult to recanalyze, has to be performed without imaging-guided navigation, normally needs the most passes and largest catheters and devices presumably leads to a higher number of IDs in these patients. Moreover, movement of the patient during or after passage of the carotid occlusion was observed in 9 patients (see selected case Fig. 2). It is well known that turning the head and neck changes the shape and curves of the carotid arteries and the turning the head maneuver has been a valuable technique in interventional neuroradiology for many years if passage of a constricted ICA is difficult.

**Fig. 3:**
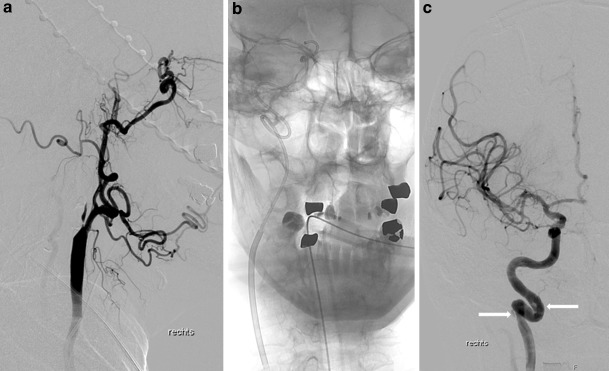
Patient 8, 79-year-old man with tandem occlusion of the internal carotid and middle cerebral arteries. **a** DSA of the right common carotid artery in lateral projection shows the pseudo-occlusion of the ICA and absence of collaterals from the branches of the external to the internal carotid artery (ECA-ICA collaterals), a typical sign of acute ICA occlusion. **b** The anterior-posterior (a.p.) view shows the tip of the 5 F aspiration catheter in front of the occluding M1 thrombus. The coiling of the ICA was passed without wire and without any problems. **c** Control DSA following recanalization of the ICA and MCA confirms two small, hemodynamically irrelevant dissections (*arrows*) of the proximal and distal segments of the looping of the distal cervical segment of the ICA

**Fig. 4:**
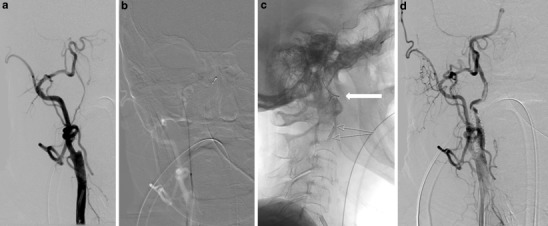
Patient 9: 79-year-old woman with right ICA/M1 tandem occlusion. **a** The stump of the occluded ICA is well outlined in the a. p. projection of the DSA. **b** Anterior-posterior roadmap with the wire in the lumen of the occluded ICA pretends a straight course up to the petrous part. **c** Change of the course of the ICA following exchange of the stiff wire and placement of a filter wire (*fine arrows*) with a soft wire tip, which allows the ICA to reshape with a kinking below the skull base (*bold arrow*). **d** Dissection following stent placement extending into the now proximally kinked cervical segment of the ICA, which was treated with a second more flexible stent

Elongation of the ICA, especially in combination with kinking and coiling, which has been shown to be associated with carotid dissections [23], became visible after recanalization of the ICA in 10 of the 16 IDs in the anterior circulation. Considering that the damage to the vessel wall is invisible until successful recanalization of the artery, we cannot establish the exact time and reason for the dissection in all our patients; however, in our experience of more than 200 carotid artery revascularizations in the setting of AIS [24], damage to the wall can happen during the passage of the wire, transit of the catheter (Fig. 3), PTA, stent placement or retrieval of instruments used to recanalize the distal tandem occlusion. Stent placement, the last step in tandem occlusion recanalization using the upside-down technique [25], was the most likely reason for carotid dissection in 2 patients. The occluded ICA appeared to be straight with the wire or distal protection material in place, but returned to the original shape with kinking or narrow looping after removal of the aspiration or distal access catheter (Fig. 4). The linear stretching of the proximal ICA segment by the stent may aggravate the kinking at the distal end of the stent, resulting in a dissection, which was treated in our 2 cases by insertion of a second, more flexible stent.

All patients with an ID-related high-grade stenosis and 3 patients with a stenosis of <80% were treated with a stent, achieving TIMI 2–3 recanalization in all but one. Stent placement in initially low-grade stenosis was performed only if control DSA indicated progression of the dissection. During the waiting period of 15–30 min, a microwire with the tip in the true lumen distal to the ID secured rapid access and stenting in the case of an increase of the false lumen and subsequent complete occlusion of the artery. Similarly, therapy in the five SWIFT ID patients consisted of conservative management in three, and stent placement and balloon angioplasty in one each [16]. The rationale for choosing the different treatment modes, recanalization rates and patient outcomes was not reported.

In our study, patients with ID were significantly more often smokers compared to patients without ID. Smoking has not been described as a risk factor for spontaneous cervicocerebral artery dissection [26]; however, in patients with spontaneous VA dissection, smokers had cerebral ischemia more often than non-smokers [27]. In smokers endothelial function is impaired, the coagulation cascade is activated, and inflammatory cytokines are upregulated [28]. Thus, an increased vulnerability of the arterial wall to mechanical injuries in smokers is possible. Patients with ID had different AIS etiologies than patients without ID. Cardioembolic strokes were less frequent in ID patients. We hypothesize that a cardioembolic occlusion of a previously normal artery may be easier to reopen than in other subtypes of stroke. Cervicocerebral arterial dissection carries the risk for serious complications, such as cerebral ischemia or subarachnoid hemorrhage. Comparing clinical outcome and mortality in the endovascularly treated AIS patients included in this study, patients with ID did not significantly differ from patients without ID. Nevertheless, two patients died as a result of AIS complications possibly related to the ID, indicating that ID is a clinically relevant complication of endovascular treatment with mechanical devices; therefore, endovascular treatment for AIS should only be performed in institutions where such complications can be managed by staff experienced in weighing the risks and benefits of this intervention and skilled in how to manage ID.

This study is mainly limited by the single center design and the retrospective identification of ID cases based on the initial report of the interventional neuroradiologist; however, the DSA series are stored in full length in our PAC System, and two senior interventional neuroradiologists independently reviewed the DSAs of all ID patients. Another limitation may be that different endovascular recanalization techniques and devices have been used since the start of this study in 2000. On the other hand, by comparing those different techniques it became evident that most IDs occur during gaining access to the thrombus, especially if an occluded, torturous internal carotid artery has to be passed without biplane roadmap navigation. Surprisingly, no ID was observed during withdrawal of a stent retriever.

This study does not allow conclusions to be drawn on how to best treat an ID. Taking into account the benign course of IDs in most patients, “watchful waiting” seems justified prior to stent implantation.

In conclusion, ID is rare in endovascular treatment of acute ischemic stroke; however, a high index of suspicion for ID is key to its detection during the endovascular intervention, to immediately provide appropriate treatment, for instance in case of progression, and thus increase the chances of a good outcome.

## Caption Electronic Supplementary Material

Supplemental online figures show the etiology of stroke and clinical outcome of the 18 patients who suffered a iatrogenic dissection during the intervention in comparison to the remaining 848 without dissection
