# Supplementary material for: Iatrogenic Vessel Dissection in Endovascular Treatment of Acute Ischemic Stroke
Source: Clin Neuroradiol. 2017 Nov 2;29(1):143–51. doi: 10.1007/s00062-017-0639-z (PMC6394531; doi:10.1007/s00062-017-0639-z)
Supplement: Supplementary file 1 — Supplemental online figures show the etiology of stroke and clinical outcome of the 18 patients who suffered a iatrogenic dissection during the intervention in comparison to the remaining 848 without dissection [file 62_2017_639_MOESM1_ESM.docx]

**Clinical Neuroradiology**

SUPPLEMENTAL ONLINE FIGURES

## Iatrogenic vessel dissection

## in endovascular treatment of acute ischemic stroke

Barbara Goeggel Simonetti, MD, Justine Hulliger, MD, Etienne Mathier, MD, Simon Jung, MD, Urs Fischer, MD MSc, Hakan Sarikaya, MD, Johannes Slotboom, PhD, Gerhard Schroth, MD, Pasquale Mordasini, MD, Jan Gralla, MD MSc, Marcel Arnold, MD

Corresponding author: Prof. Dr. med. Gerhard Schroth, Institute of Diagnostic and Interventional Neuroradiology, University Hospital Bern, Freiburgstrasse 10, 3010 Bern, Switzerland.

E-mail: [Gerhard.Schroth@insel.ch](mailto:Gerhard.Schroth@insel.ch)

**On-line Fig. 1:** AIS-etiology according to TOAST in patients with ID (n=18) and patients without ID (n=848)


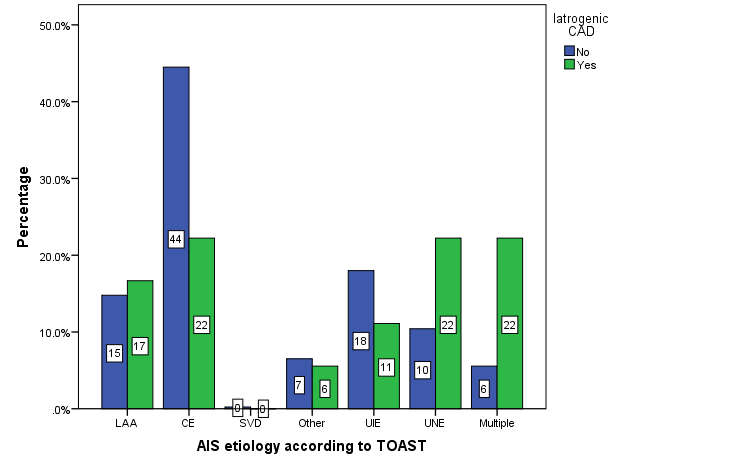


*p*=0.041

Abbreviations: LAA = large-artery atherosclerosis. CE = cardioembolism. SVD = small-vessel disease. UNE = negative evaluation. UIE = incomplete evaluation. Multiple = more than one etiology possible.

**On-line Fig. 2:** Outcome of patients with ID (n=18) versus patients without iatrogenic CAD (n=848)


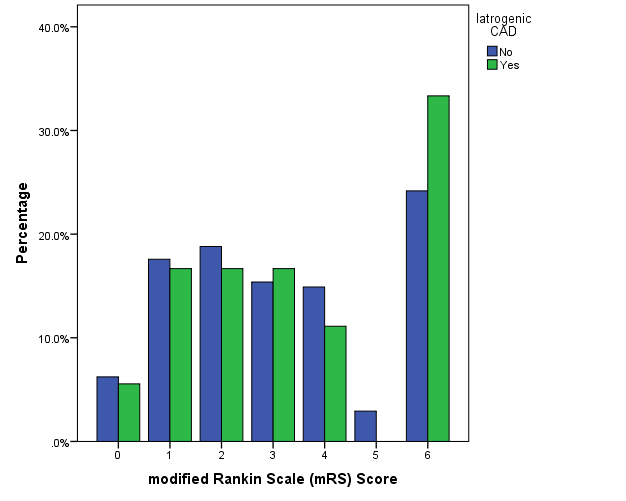


*p*=0.639
